# Decreased plasma levels of soluble CD18 link leukocyte infiltration with disease activity in spondyloarthritis

**DOI:** 10.1186/ar4471

**Published:** 2014-02-04

**Authors:** Tue W Kragstrup, Babak Jalilian, Malene Hvid, Anders Kjærgaard, René Østgård, Berit Schiøttz-Christensen, Anne G Jurik, William H Robinson, Thomas Vorup-Jensen, Bent Deleuran

**Affiliations:** 1 Department of Biomedicine, Aarhus University, Wilhelm Meyers Allé 4, DK-8000 Aarhus, Denmark; 2 Division of Immunology and Rheumatology, Stanford University, 296 Campus Drive, Stanford, CA 94305, USA; 3 Department of Clinical Medicine, Aarhus University, Brendstrupgårdsvej 100, DK-8200 Aarhus N Denmark; 4 Department of Anaesthesiology, Aarhus University Hospital, Aarhus, Denmark; 5 Aarhus Rheumatology Clinic, Skt. Clemens Torv 17, DK-8000 Denmark; 6 Department of Radiology, Aarhus University Hospital, Aarhus, Denmark; 7 Department of Rheumatology, Aarhus University Hospital, Aarhus, Denmark

## Abstract

**Introduction:**

Spondyloarthritis (SpA) comprises a group of diseases often associated with HLA-B27 and characterized by inflammation of the entheses and joints of the axial skeleton. The inflammatory process in SpA is presumably driven by innate immune cells but is still poorly understood. Thus, new tools for monitoring and treating inflammation are needed. The family of CD18 integrins is pivotal in guiding leukocytes to sites of inflammation, and CD18 hypomorphic mice develop a disease resembling SpA. Previously, we demonstrated that altered soluble CD18 (sCD18) complexes in the blood and synovial fluid of patients with arthritis have anti-inflammatory functions. Here, we study the mechanisms for these alterations and their association with SpA disease activity.

**Methods:**

Plasma levels of sCD18 in a study population with 84 patients with SpA and matched healthy controls were analyzed with a time-resolved immunoflourometric assay (TRIFMA). Binding of sCD18 to endothelial cells and fibroblast-like synoviocytes (FLSs) was studied with confocal microscopy. Shedding of CD18 from peripheral blood mononuclear cells (PBMCs) was studied with flow cytometry and TRIFMA.

**Results:**

Plasma levels of sCD18 were decreased in patients with SpA compared with healthy volunteers (*P* <0.001), and the lowest levels were in the HLA-B27-positive subgroup (*P* <0.05). In a multiple regression model, the sCD18 levels exhibited an inverse correlation with the Bath Ankylosing Spondylitis Disease Activity Index (BASDAI) (*P* <0.05), the level of morning stiffness (*P* <0.05), the Bath Ankylosing Spondilitis Metrology Index (*P* <0.05), the physician global assessment score (*P* <0.01), and the sacroiliac magnetic resonance imaging activity score (*P* <0.05). The mechanisms for these changes could be simulated *in vitro*. First, sCD18 in plasma adhered to inflammation-induced intercellular adhesion molecule 1 (ICAM-1) on endothelial cells and FLS, indicating increased consumption. Second, CD18 shedding from SpA PBMCs correlated inversely with the BASDAI (*P* <0.05), suggesting insufficient generation. CD18 was shed primarily from intermediate CD14^++^ CD16^+^ monocytes, supporting the view that alterations in innate immunity can regulate the inflammatory processes in SpA.

**Conclusions:**

Taken together, the failure of patients with SpA to maintain adequate sCD18 levels may reflect insufficient CD18 shedding from monocytes to counterbalance the capture of sCD18 complexes to inflammation-induced ICAM-1. This could increase the availability of ICAM-1 molecules on the endothelium and in the synovium, facilitating leukocyte migration to the entheses and joints and aggregating disease activity.

## Introduction

Spondyloarthritis (SpA) comprises a group of common inflammatory musculoskeletal diseases, including ankylosing spondylitis, psoriatic arthritis, arthritis associated with inflammatory bowel disease, reactive arthritis, and undifferentiated SpA. Patients with SpA share the characteristics of a high frequency of the HLA-B27 allele in the MHC I loci and disease affecting the entheses and joints of the axial skeleton [1]. In SpA, disease disability is the sum of the inflammatory burden and chronic changes. Thus, distinguishing between inflammatory activity and chronic problems is critical for choosing a proper clinical intervention [2,3].

The inflammatory process in SpA involves innate immune cells such as macrophages, monocytes, and fibroblasts [4]. Monocytes are present in the blood and can be divided into the three subsets: CD14^++^ CD16^-^ classic monocytes, CD14^++^ CD16^+^ intermediate monocytes, and CD14^+^ CD16^++^ non-classic monocytes [5]. The intermediate monocyte subset increases during an infection [6] and is elevated in RA and SpA [7-9]. Intermediate monocytes have been described to have both pro-inflammatory and anti-inflammatory activities [5], and the role of intermediate monocytes in arthritis is still elusive. Disease activity in SpA is associated with migration of leukocytes to inflamed joints and entheses [1]. However, the complexity of the inflammatory process in SpA is still poorly understood.

The β2 (CD18) family of integrins includes CD11a/CD18 (LFA-1), CD11b/CD18 (Mac-1 or complement receptor [CR] 3), CD11c/CD18 (CR4, p150,95), and CD11d/CD18 and is crucial in transendothelial migration by the binding of CD11a/CD18 to intercellular adhesion molecule 1 (ICAM-1) [10,11]. In arthritis, the increased expression of ICAM-1 results in the migration of leukocytes to the joints [12]. Animal studies support a central role of CD18 in the development of SpA. The mouse PL/J strain carries the CD18 hypomorphic mutation, which reduces the expression of CD18 to 2% to 16% of wild-type levels [13]. These mice develop a skin disease that closely resembles human psoriasis together with a condition very similar to psoriatic arthritis dominated by enthesitis [14], and the inflammatory response critically involves macrophages and T helper 17 (Th17) cells [15,16]. Tumor necrosis factor-alpha (TNFα) induces endothelial expression of ICAM-1 as well as activation of integrins for ligand binding [17]. In this way, the success of biological therapy with anti-TNFα in the treatment of SpA also indirectly suggests that the CD18 integrins play a role in human disease.

Emerging literature [18-21] has identified soluble complexes of CD11/CD18. These complexes bind ICAM-1 [18,19] in a way that competes with the binding of cellular-expressed CD11/CD18, implying an anti-inflammatory role [18]. In humans, we found alterations in the soluble CD18 (sCD18) plasma concentrations in patients with rheumatoid arthritis (RA) or SpA whereas patients with osteoarthritis had no such changes [18]. Shedding of CD18 is increased by TNFα stimulation [18] and occurs in processes that involve matrix metalloproteinase (MMP)-3 and MMP-9 [22,23]. MMP-3 is the activator of the MMP-9 zymogen [24], and dysregulation is associated with a wide variety of autoimmune disorders [25], including RA [26] and SpA [27]. Recently, comprehensive studies in transgenic animals showed that leukocyte migration is critically dependent on efficient CD18 shedding, notably in the case of macrophage efflux from zones of inflammation [22]. However, not much is known about the cellular sources and targets of sCD18, and the association of sCD18 levels in the blood with other disease activity parameters has never been studied in any disease.

We previously demonstrated alterations in sCD18 complexes in the blood and synovial fluid of patients with arthritis and showed that sCD18 binding to ICAM-1 could be an anti-inflammatory regulatory mechanism in the immune system. Here, we study the mechanisms for these alterations and their association with SpA disease activity. In SpA, low sCD18 concentrations were part of an aggravated disease activity state. The low sCD18 levels seemed to be a result of a combination of increased capture of sCD11/CD18 complexes onto ICAM-1 expressed on inflamed tissues and insufficient CD18 shedding from monocytes. Thus, the resulting increased availability of ICAM-1 molecules on the endothelium and in the synovium could facilitate increased leukocyte infiltration in the entheses and joints and increased disease activity. Hereby, our findings link regulation of leukocyte infiltration by innate immune cells with inflammation and disease activity in SpA.

## Materials and methods

### Study populations

At the time of inclusion, the study population comprised 84 patients with axial SpA according to the European Spondyloarthropathy Study Group (ESSG) criteria [28,29]. At a follow-up 4 years later, 43 of these patients were examined again (Table 1). A total of 47 patients met the modified New York criteria for ankylosing spondylitis at the time of inclusion [28,29], whereas 37 patients had non-radiograhic SpA. The study population was clinically well characterized, and self-assessment scores and clinical scores comprised physician global assessment visual analogue scale (VAS), patient pain VAS, patient global VAS, Bath Ankylosing Spondylitis Disease Activity Index (BASDAI), Bath Ankylosing Spondylitis Functional Index (BASFI), Bath Ankylosing Spondilitis Metrology Index (BASMI), thoracic chest expansion and disease duration at both baseline and follow-up. The test results included C-reactive protein (CRP), HLA-B27 status, radiography, and magnetic resonance imaging (MRI). MRI of the sacroiliac joints (SIJs) and the entire spine at both baseline and follow-up and radiography of the SIJ at baseline were included in this study (Table 1). A specialist radiologist who was experienced in SpA but who had no knowledge of the clinical findings analyzed all MRI and radiographic tests. The MRI changes were graded by using methods previously described [30,31]. In summary, the MRI SIJ and spinal activity scores were made in accordance with the Danish methods primarily differing from the Spondyloarthritis Research Consortium of Canada (SPARCC) methods by using fully three-dimensional assessment of the SIJ and the spine [32,33]. The intra- and inter-observer agreements of the methods have been demonstrated to be acceptable [30,31].

**Table 1:** Patient characteristics

**Characteristics**	**At time of inclusion**	**At follow-up**	**HCs**
	**(n = 84)**	**(n = 43)**	**(n = 28)**
**Age in years (mean)**	37 (35-39)	41 (38-43)	42.9
**Gender, percentage female**	58	59	57
**Diagnosis, percentage of patients**			
**Ankylosing spondylitis**	19	19	-
**Psoriatic arthritis**	11	12	-
**Enteropathic arthritis**	4	5	-
**Reactive arthritis**	15	17	-
**Undifferentiated SpA**	51	47	-
**HLA-B27, percentage positive**	60	55	-
**Disease duration in years (mean)**^**a**^	8.0 (7.0-9.0)	12.6 (9.8-15)	-
**Treatment, percentage of patients**			
**No**	67	-	-
**MTX**	8	-	-
**Salazopyrin**	11	-	-
**Anti-TNFα**	7	12	-
**Self-assessment scores**			-
**BASDAI, 0-100 (mean)**	32 (26-37)	35 (28-42)	-
**BASFI, 0-100 (mean)**	20 (16-25)	22 (16-28)	-
**Patient pain, 0-100 (mean)**	32 (26-38)	33 (26-41)	-
**Patient global, 0-100 (mean)**	32 (26-38)	31 (23-38)	-
**Level of morning stiffness, 0-100 (mean)**	36 (29-43)	36 (27-44)	-
**Duration of morning stiffness, 0-100 (mean)**	33 (26-41)	29 (20-38)	-
**Clinical scores**			
**BASMI, 0-100 (median)**	0 (0-0)	0 (0-10)	-
**Physician global, 0-100 (mean)**	16 (13-20)	20 (16-25)	-
**Thoracic chest expansion in cm (mean)**	4.1 (3.9-4.4)	4.9 (4.5-5.3)	-
**Test results**			
**CRP in mg/L (median)**	2.1 (1.3-3.9)	1.0 (0.5-2.5)	
**SIJ MRI activity, 0-40 (mean)**	7.5 (5.9-9.0)	4.3 (2.9-5.7)	-
**Spine MRI activity, 0-81 (median)**	1 (0-4)	1 (0-2)	-
**SIJ MRI chronicity, 0-48 (mean)**	15 (12-18)	17 (13-22)	-
**Spine MRI chronicity, 0-207 (median)**	0 (0-4)	0 (0-5)	-

Numbers in parentheses are 95% confidence interval for measures with a Gaussian distribution and 25% to 75% percentiles for measures with a non-Gaussian distribution. ^a^Disease duration was defined as duration since start of symptoms. -, not available; Anti-TNF-α, anti-tumor necrosis factor-alpha; BASDAI, Bath Ankylosing Spondylitis Disease Activity Index; BASFI, Bath Ankylosing Spondylitis Functional Index; BASMI, Bath Ankylosing Spondilitis Metrology Index; CRP, C-reactive protein; HC, healthy control; MRI, magnetic resonance imaging; MTX, methotrexate; SIJ, sacroiliac joint; SpA, spondyloarthritis.

One SpA patient with peripheral arthritis was included for growing fibroblast-like synoviocytes (FLSs) from synovial fluid mononuclear cells (SFMCs). The patient contacted the out-patient clinic because of a knee joint effusion. No disease activity or prognosis scores or test results were recorded.

Plasma from age- and gender-matched healthy controls (HCs) from either the Blood Donor Bank at Aarhus University Hospital or from patients undergoing orthopedic surgery at the Department of Orthopaedics at Aarhus University Hospital (n = 28) was included for measuring sCD18 plasma concentration (Table 1). Peripheral blood mononuclear cells (PBMCs) from HCs were included for *in vitro* studies of spontaneous shedding of CD18 from PBMCs, T cells, natural killer (NK) cells, and monocytes.

All plasma samples were collected in heparinized tubes and kept at -80°C until use. PBMCs and SFMCs were isolated by conventional Ficoll-Paque (GE Healthcare, Little Chalfont, Buckinghamshire, UK) density-gradient centrifugation and cryopreserved at -135°C until time of analysis. HC PBMCs were isolated from buffy coats for the *in vitro* studies of spontaneous shedding of CD18.

### Quantification of sCD18 and MMP-9 by time-resolved immunofluorometric assays

The IgG1 monoclonal antibodies (mAbs) against CD18 were produced by GenScript from the hybridoma cell lines CRL-2839 (KIM185) and CRL-2838 (KIM127) followed by protein A/G purification. The mouse IgG1 isotype (cat. no. M7894; Sigma-Aldrich, St. Louis, MO, USA) was purified from an ascites suspension with protein A/G purification. Biotinylated forms of the antibodies were made by use of Biotin *N*-hydroxysuccinimide ester (cat. no. 14405; Sigma-Aldrich) [18].

Detection of sCD18 in plasma samples and supernatants from *in vitro* cell cultures was carried out by time-resolved immunofluorometric assays (TRIFMAs) by using a sandwich technique as previously described [18]. Briefly, microtiter wells (FluoroNunc Maxisorp, 437958; Nunc, Roskilde, Denmark) were coated with antibody to CD18 (KIM185) or mouse IgG1. The wells were washed in Tris-buffered saline (TBS)/Tween and blocked by incubation with 200 μL TBS with 1 mg/mL human serum albumin for 1 hour at room temperature (RT). After washes, samples of 100 μL heparinized plasma diluted 1:10 and 1:5 or supernatants diluted 1:2 in TBS/Tween with 1 mM CaCl_2_, 1 mM MgCl_2_, and 100 μg/mL aggregated human Ig were added to the wells, and the plates were incubated overnight at 4°C. After incubation of the diluted samples, the wells were washed and subsequently incubated with 100 μL biotinylated antibody to CD18 (KIM127). After washing of the wells, Eu^3+^-conjugated streptavidin was applied and the signals were read by time-resolved fluorometry. Signals from patient samples were compared against a standard curve made from titrations of plasma from an HC.

Detection of MMP-9 was carried out by TRIFMA as described for the quantification of sCD18 by modifying a commercially available enzyme-linked immunosorbent assay kit (SEK10327; Sino Biological, Inc., Daxing, China). An IgG1 mAb to MMP-9 (clone 4A4H5D8) was coated into microtiter plate wells at a concentration of 2.0 μg/mL. Samples were diluted in TBS/Tween with CaCl_2_, MgCl_2_, and aggregated human Ig. For detection of captured MMP-9 antigen, a rabbit mAb to MMP-9 (clone 31) was diluted to a concentration of 1 μg/mL in TBS/Tween with 100 μg/mL bovine IgG [18]. Signals from the analyzed samples were compared against a standard curve made from titrations of the HC plasma also used for sCD18 measurements. As a control, plates coated with murine IgG1 isotype were prepared and the signals from these wells were subtracted from the signals recorded in wells with the mAb to MMP-9.

### Confocal microscopy of sCD18 binding to ICAM-1 expressed on EA.hy926 cells and SpA FLSs

The EA.hy926 endothelial cell line was cultured in Dulbecco’s modified Eagle’s medium (DMEM) supplemented with 10% (vol/vol) fetal calf serum (FCS), penicillin, streptomycin, and glutamine. FLSs were grown from SFMCs as done previously [34]. Briefly, SFMCs were isolated and cryopreserved as described above. The cells were thawed and cultured in supplemented DMEM at a concentration of 2 × 10^6^ cells/mL at 37°C in a humidified incubator with 5% CO_2_ (vol/vol) replacing the medium every 3 to 4 days. When the cell layer was 70% confluent, the FLSs were passaged by trypsin/ethylenediaminetetraacetic acid (EDTA) treatment and used for analyses at passage 5. Sterile glass slides were placed in 24-well cell culture plates and aseptically treated with 300 μL of 0.1% (wt/vol) polylysine dissolved in water (cat. no. P8920; Sigma-Aldrich) for 5 minutes. After thorough rinsing with sterile water, the glass slides were allowed to dry for 2 hours. Either EA.hy926 cells or FLSs were then seeded at a concentration of 2.5 × 10^4^ cells/mL in supplemented DMEM and incubated for 24 hours at 37°C. The cells were either stimulated with TNFα (cat. no. 300-01A; PeproTech, Rocky Hill, NJ, USA) at a concentration of 10 ng/mL or incubated with medium alone for 24 hours at 37°C. After aspiration of the culture medium, cells were incubated with 50% (vol/vol) heat-inactivated HC serum with a known concentration of sCD11/CD18 (542 mU sCD18 per mL) in 150 mM NaCl, 5 mM KCl, 1 mM MgCl_2_, 1.8 mM CaCl_2_, 1 mM MnCl_2_, 10 mM HEPES, pH 7.4 (Binding Buffer). As another source of sCD11/CD18, additional incubations were made with 50% (vol/vol) SFMC culture supernatant with sCD11/CD18 (approximately 25 mU/mL) in Binding Buffer or Binding Buffer alone for 16 hours at 37°C. Wells were washed twice, and cells were fixed with 300 μL 3.7% (vol/vol) paraformaldehyde for 10 minutes at RT. Unspecific binding was blocked with both 10 μg/mL murine IgG and 10 μg/mL bovine IgG in 200 μL Binding Buffer for 30 minutes at RT. Cells were stained with either biotinylated mouse IgG1 anti-CD18 (KIM127) or biotinylated mouse IgG1 isotype in combination with streptavidin Alexa flour 546 (cat. no. S11225; Invitrogen) or mouse IgG1 anti-ICAM-1 fluorescein isothiocyanate (FITC) antibody (clone 6.5B5, cat. no. F7143; Dako, Glostrup, Denmark). Wells were washed twice, and glass slides were placed in 2 μL of anti-fade mounting medium with 1/1,000 4′,6-diamidino-2-phenylindole (DAPI) (cat. no. D9542; Sigma-Aldrich) and allowed to dry overnight. All micrographs were collected by using a Zeiss LSM-710 confocal microscope and Pixelmator (Pixelmator Team Ltd., Vilinius, Lithuania) for editing.

### Flow cytometric analyses of PBMCs

Cells were transferred to FACS tubes (Nunc) and blocked for unspecific binding in phosphate-buffered saline with 0.5% (wt/vol) bovine serum albumin (cat. no. 12659; Calbiochem, now part of EMD Biosciences, Inc., San Diego, CA, USA), 0.09% (wt/vol) NaN_3_ together with 10 μg/mL murine IgG and 10 μg/mL bovine IgG (cat. nos. 015-000-003 and 001-000-003; Jackson ImmunoResearch, West Grove, PA, USA). Cells were stained with either biotinylated mouse IgG1 mAb to CD18 (KIM185) or biotinylated mouse IgG1 isotypic control in combination with streptavidin-FITC (Dako). According to the manufacturer’s instructions, mouse IgG1 anti-CD163 PE antibody (clone MAC2-158, cat. no. CD163-158P; Trillium Diagnostics), mouse IgG2a anti-CD14 ECD antibody (clone RMO52, cat. no. PN IM2707U; Beckman Coulter), mouse IgG1 anti-CD16 APC antibody (clone eBioCB16, cat. no. 17-0168; eBioscience, San Diego, CA, USA), mouse IgG1 anti-CD3 ECD antibody (clone UCHT1, cat. no. A07748; Immunotech, Beckman Coulter), and mouse IgG2b anti-CD56 antibody (clone c5.9, cat. no. R7251; Dako) were used for subanalysis of the PBMCs, and the Live/Dead staining (cat. no. L10119; Invitrogen) was used for viability. All samples were analyzed within 24 hours by using an FC500 with CXP software (Beckman Coulter) and FlowJo software version 9.6 (Tree Star Inc., Ashland, OR, USA).

### *In vitro* culture experiments with PBMCs

For *in vitro* culture experiments with PBMCs, the cells were thawed and cultured in RPMI medium supplemented with 10% (vol/vol) FCS, penicillin, streptomycin, and glutamine. To study differences in spontaneous CD18 shedding between SpA and HC unfractionated PBMCs, the cells were incubated at a concentration of 1 × 10^6^ cells/mL. To study differences in spontaneous CD18 shedding from PBMC subsets, T cells (cat. no. 130-091-156; Miltenyi Biotec, Bergisch Gladbach, Germany), NK cells (cat. no. 130-092-657; Miltenyi Biotec), and monocytes (cat. no. 130-091-153; Miltenyi Biotec) were isolated by negative selection in accordance with the instructions of the manufacturer and incubated at a concentration of 1 × 10^6^ cells/mL. In all PBMC experiments, cells were cultured for 48 hours at 37°C in a humidified incubator 5% (vol/vol) CO_2_ without changing of medium. After incubation, supernatants were stored frozen at -80°C for later sCD18 analysis with TRIFMA.

### Statistical analyses

Gaussian distributed measures were presented by the mean value, whereas measures with a non-Gaussian distribution were presented by the median value as indicated. Prior to analyses for statistical significance, the plasma sCD18 levels were log-transformed. Comparisons of the plasma sCD18 levels between groups of unpaired and paired samples were made by using the Student *t* test in the unpaired and paired modes, respectively. Correlation analyses between disease activity parameters and the plasma sCD18 levels were performed with the Pearson correlation for measures with a Gaussian distribution and with the Spearman correlation for measures with a non-Gaussian distribution. Multiple regression models were made with the plasma sCD18 levels and disease activity parameters correcting for age, disease duration, HLA-B27 status, and treatment at time of inclusion. The same analyses were made after additional correction for CRP. All cell culture experiments were analyzed with non-parametric statistics. The Mann-Whitney *U* test was used for unpaired comparisons, the Wilcoxon signed rank test was used for paired data, and the Spearman correlation was used for association studies. A two-tailed *P* value below 0.05 was considered significant. Calculations and graphs were made with Stata version 11.1 (StataCorp LP, College Station, TX, USA) and GraphPad Prism version 5 (GraphPad Software, San Diego, CA, USA).

### Approval of studies using human samples

All SpA samples were collected in the out-patient clinic in Aarhus University Hospital or in the Aarhus Rheumatology Clinic private practice. All samples were obtained after informed written consent according to the Declaration of Helsinki and approved by the Research Ethics Committees of Central Jutland (project numbers 20050046 and 20058432) and the Danish Data Protection Agency.

## Results

### The plasma level of sCD18 was lower in SpA patients compared with healthy controls

**Figure 1:**
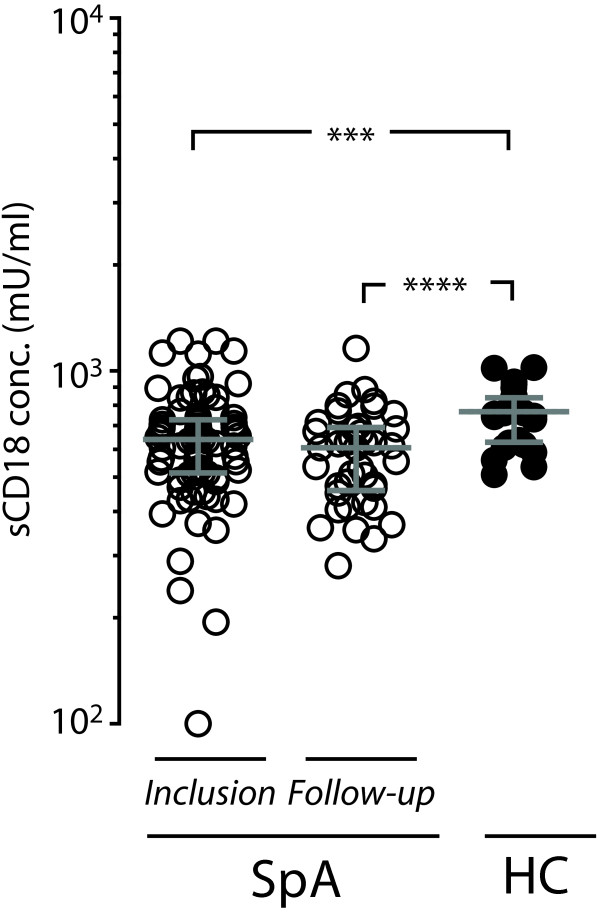
**Plasma concentrations of soluble CD18 (sCD18) in patients with spondyloarthritis (SpA) at the time of inclusion and at the 4-year follow-up and healthy controls (HCs).** The median values of plasma sCD18 were 639.7 mU/mL (interquartile range 514.3-726.8 mU/mL) for patients with SpA at the time of inclusion, 606.1 mU/mL (interquartile range 458.4-691.5 mU/mL) for patients with SpA at the 4-year follow-up, and 767.2 mU/mL (interquartile range 628.1-839.7 mU/mL) for HCs. Bars indicate median and interquartile range. ***P <0.001, ****P <0.0001.

We measured the concentration of sCD18 in plasma samples from patients with SpA and HCs by using TRIFMA. The levels of sCD18 in plasma from patients with SpA at both time of inclusion and 4-year follow-up were significantly lower than in plasma from HCs (Figure 1). Furthermore, the levels of sCD18 showed a clear overlap between the three groups. There was no significant difference between the plasma sCD18 levels in patients with SpA at time of inclusion compared with the second time-point 4 years later (*P* = 0.10) (Figure 1). No associations were observed between gender or age and plasma sCD18 levels for either patients with SpA or HCs.

### The plasma level of sCD18 was negatively associated with disease activity and decreased in HLA-B27-positive SpA

We correlated the sCD18 concentration with several disease activity parameters to analyze for an association between decrease in sCD18 plasma level and disease activity. These included scores calculated from information provided by the patients (BASDAI, BASFI, patient pain VAS, patient global assessment score, and morning stiffness), scores resulting from clinical examination (BASMI, physician global assessment score, and thoracic chest expansion), and objective measures (CRP, sacroiliac joint [SIJ] MRI activity score, and spine MRI activity score).

Significant negative correlations were observed between the plasma sCD18 levels and BASMI (r = -0.25, *P* <0.05), physician global assessment score (r = -0.34, *P* <0.01), CRP (r = -0.34, *P* <0.01), and SIJ MRI activity score (r = -0.25, *P* <0.05). No correlations were observed between sCD18 and MRI chronicity scores.

**Figure 2:**
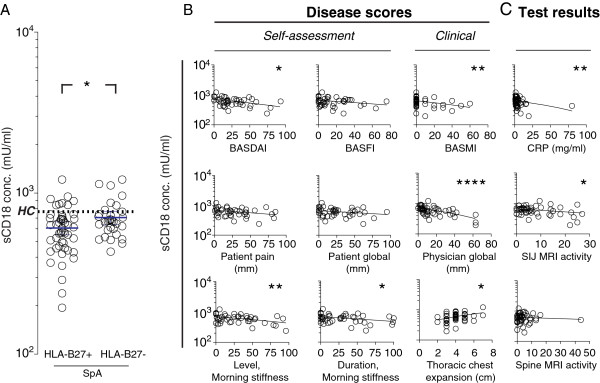
**The soluble CD18 (sCD18) plasma concentration, HLA genotype, and spondyloarthritis (SpA) disease activity. (A)** The sCD18 plasma level in HLA-B27-positive versus HLA-B27-negative SpA patients at the time of inclusion. The median values were 605.7 mU/mL (interquartile range 461.6-704.1 mU/mL) for HLA-B27-positive SpA patients and 670.0 mU/mL (interquartile range 591.8-760.1 mU/mL) for HLA-B27-negative SpA patients. Bars indicate median and interquartile range. **(B)** Correlation between the sCD18 plasma levels in HLA-B27-positive SpA patients and self-assessment and clinical scores. **(C)** Correlation between the sCD18 plasma levels in HLA-B27-positive SpA patients and clinical test results. Solid lines represent the best fit in linear regression. *P <0.05; **P <0.01; ***P <0.001; ****P <0.0001.

The association of sCD18 plasma level and disease activity was further investigated by using subgroup and multivariate analyses. Overall, the levels of sCD18 in plasma from HLA-B27-positive SpA patients were reduced compared with HLA-B27-negative SpA patients (Figure 2A). No differences in plasma sCD18 levels were observed when comparing the SpA subtypes (ankylosing spondylitis, psoriatic arthritis, enteropathic arthritis, reactive arthritis, and undifferentiated SpA), when comparing patients fulfilling or not fulfilling the modified New York criteria for ankylosing spondylitis, or when comparing patients treated or not treated with a TNFα inhibitor. Because HLA-B27 positivity adds to the strength of the SpA diagnosis and because we found an association between the sCD18 plasma concentration and HLA-B27 positivity, the HLA-B27-positive SpA patients were analyzed separately. In this subgroup, significant negative correlations were present between the plasma sCD18 levels and BASDAI (r = -0.39), BASMI (r = -0.38), level of morning stiffness (r = -0.43), duration of morning stiffness (r = -0.33), physician global assessment score (r = -0.62), CRP (r = -0.38), and SIJ MRI activity score (r = -0.35) while thoracic chest expansion showed a positive association to the plasma sCD18 level (r = 0.37) (Figure 2B and C).

The association between plasma sCD18 levels and markers of disease activity was also tested in regression models with correction for age, disease duration, HLA-B27 status, and treatment at time of inclusion. In these models, significant negative correlations were observed between the plasma sCD18 levels and BASDAI, level of morning stiffness, physician global assessment score, BASMI, and SIJ MRI activity score (Tables 2 and 3). Furthermore, we analyzed these associations after correcting for CRP to evaluate whether sCD18 adds to the information already achieved by the CRP. The associations remained significant for BASDAI, level of morning stiffness, BASMI, and physician global assessment score (Additional file 1: Table S1 and Additional file 2: Table S2). The same tendencies were seen between plasma sCD18 and markers of disease activity at 4-year follow-up, but these were not significant (data not shown).

**Table 2:** Associations at time of inclusion between plasma soluble CD18 levels in all spondyloarthritis patients and self-assessment scores after correction for age, disease duration, HLA-B27 status, and treatment

		**BASDAI**	**BASFI**	**Patient pain**	**Patient global**	**Morning stiffness, level**	**Morning stiffness, duration**
**sCD18**	r^a^	**-0.29**	**-**0.083	**-**0.14	**-**0.11	**-0.27**	**-**0.15
	*P*	**0.026**	0.54	0.30	0.42	**0.042**	0.26

Bold numbers indicate significant correlations. ^a^Partial correlation coefficients. BASDAI, Bath Ankylosing Spondylitis Disease Activity Index; BASFI, Bath Ankylosing Spondylitis Functional Index; sCD18, soluble CD18.

**Table 3:** Associations at time of inclusion between plasma soluble CD18 levels in all spondyloarthritis patients and clinical scores and test results after correction for age, disease duration, HLA-B27 status, and treatment.

		**BASMI**	**Physician global**	**Thoracic chest expansion**	**CRP**	**SIJ activity**	**Spine activity**
**sCD18**	r^a^	**-0.030**	**-0.42**	0.12	-0.23	**-0.27**	-0.068
	*P*	**0.015**	**0.001**	0.34	0.069	**0.032**	0.59

Bold numbers indicate significant associations. ^a^Partial correlation coefficients. BASMI, Bath Ankylosing Spondilitis Metrology Index; CRP, C-reactive protein; sCD18, soluble CD18; SIJ, sacroiliac joint.

The value of sCD18 plasma concentration as a prognostic marker in SpA was tested by correlating the plasma sCD18 concentration at baseline with disease activity scores at the 4-year follow-up (BASDAI, BASFI, patient pain VAS, patient global assessment score, morning stiffness, BASMI, physician global assessment score, thoracic chest expansion, CRP, SIJ MRI activity score, and spine MRI activity score) and changes in these parameters from the inclusion to the 4-year follow-up. When the entire group of SpA patients was examined, there were no significant associations. When only the HLA-B27-positive SpA patients were examined, there was a significant negative correlation between the plasma sCD18 levels at time of inclusion and disease activity at 4-year follow-up measured by BASDAI (r = -0.42, *P* <0.05).

### sCD11/CD18 complexes were captured by ICAM-1 on EA.hy926 cells and SpA FLSs

**Figure 3:**
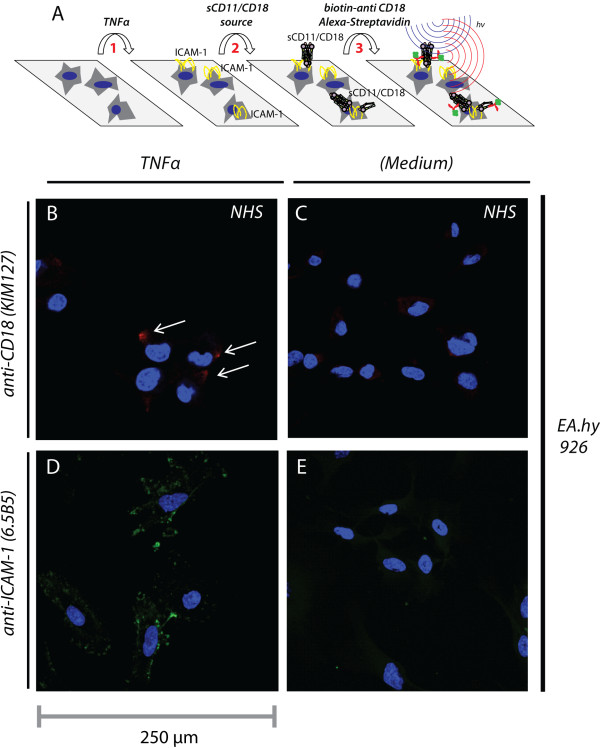
**Confocal microscopy analysis of the ability of sCD11/CD18 complexes to bind intercellular adhesion molecule 1 (ICAM-1) expressed on the human umbilical vein cell line EA.hy926. (A)** Illustration of the cellular incubations. In step 1, adherent cells were stimulated with 10 ng/mL tumor necrosis factor-alpha (TNFα), which increased the ICAM-1 expression. In step 2, a source of CD11/CD18 was added (that is, either normal human serum (NHS) or supernatant from synovial fluid mononuclear cell culture). In step 3, biotinylated antibody recognizing ligand-binding activated CD11/CD18 (KIM127) was added followed by addition of fluorochrom-labelled streptavidin for detection using confocal microscopy. Binding of sCD18 to ICAM-1 expressed on EA.hy926 cells incubated with **(B)** or without **(C)** TNFα. Red staining indicates the binding of sCD18, further indicated with white arrows. The positions of cell nuclei were located by 4′,6-diamidino-2-phenylindole (DAPI) staining, indicated in blue. The staining was distinctly localized to small foci on the cell membrane on 10% to 15% of the cells. Expression of ICAM-1 on EA.hy926 cells incubated with **(D)** or without **(E)** TNFα. ICAM-1 is indicated with a green staining, and cell nuclei are indicated with a blue staining.

To provide a rationale for the negative relation between plasma sCD18 levels and disease activity, the ability of sCD18 to bind inflammation-induced ICAM-1 was studied as outlined in the illustration (Figure 3A). First, cells were stimulated with TNFα to increase the ICAM-1 expression in the membrane. Second, incubations were made with sCD18. Third, the cells were stained for captured sCD18.

A clear signal for sCD18 capture was obtained when staining the endothelial cell line EA.hy926 after stimulation of the cells with TNFα and incubation with normal human serum (NHS) as the sCD18 source (Figure 3B). In the absence of TNFα stimulation, no staining was observed (Figure 3C). As further controls, the cells were incubated with medium without sCD18 or stained with isotype mouse IgG1. In these conditions, no staining was seen confirming the CD18 specificity of the KIM127 antibody in our experiments (Additional file 3: Figure S1). Similar findings were made when using primary SpA FLS and when using SFMC supernatant as sCD18 provider (Additional file 3: Figure S1).

The cells presented an uneven pattern of ICAM-1 expression across the cell membrane (Figure 3D) similar to observations made by Carman and colleagues on human umbilical vein endothelial cells [35,36]. The strong ICAM-1 expression was not found in the absence of TNFα treatment (Figure 3E), suggesting that such strong expression was required for sCD18 capture. ICAM-1 foci with a particularly strong staining was found on the perimeter of the cell as judged from the distance (approximately 16-20 μm) to the cell nucleus (Figure 3D). The distribution of CD18 staining on EA.hy 926 cells showed a similar staining pattern (Figure 3B). We examined the capture of sCD18 from the supernatants quantitatively to evaluate whether this weak and distinct staining could be explained by stochiometric shortage of sCD11/CD18. Supernatants containing 50% (vol/vol) NHS or 50% (vol/vol) SFMC supernatant were harvested after 16 hours of incubation with cells pre-treated either with TNFα or without such treatment. Only a small reduction in the sCD18 concentration of the supernatants was found after the cellular incubation, suggesting that the highly localized staining was not explained by stochiometric shortage of sCD11/CD18 (Additional file 4: Figure S2).

### CD18 shedding from PBMCs was affected by donor disease activity

**Figure 4:**
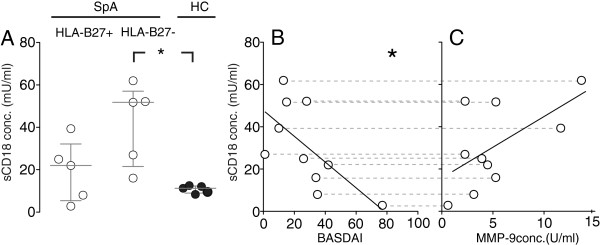
**Spontaneous shedding of CD18 from peripheral blood mononuclear cells (PBMCs) cultured *in vitro*. (A)** Levels of spontaneous shedding of CD18 from spondyloarthritis (SpA) and healthy control (HC) PBMCs. The median values were 22.0 mU/mL (interquartile range 14.0-51.8 mU/mL) for HLA-B27-positive SpA patients, 51.7 mU/mL (interquartile range 21.5-57.0 mU/mL) for HLA-B27-negative SpA patients, and 11.2 mU/mL (interquartile range 8.59-12.3 mU/mL) for HCs. PBMCs from 5 HLA-B27-positive SpA patients, 5 HLA-B27-negative patients, and 5 HCs were used. Bars indicate median and interquartile range. **(B)** Correlation between PBMC donor BASDAI score and the spontaneously shed CD18 in the PBMC culture supernatant. **(C)** CD18 spontaneous shedding capacity and correlation with matrix metalloproteinase-9 (MMP-9) production. PBMCs from 10 patients with SpA were used. Hatched horizontal lines connect identical measurements of sCD18. Solid black lines represent the best fit in linear regression. *P <0.05.

To further analyze the negative relation between plasma sCD18 levels and disease activity, the CD18 shedding was studied in cultures with PBMCs derived from patients with SpA or HCs. In a simple comparison, the spontaneous shedding of CD18 from PBMCs derived from patients with SpA was increased compared with cultures of PBMCs from HCs (*P* <0.01). In subgroup analysis, the spontaneous shedding of CD18 from PBMCs derived from HLA-B27-negative SpA patients was increased compared with cultures of PBMCs from HCs (Figure 4A), but there were no differences between HLA-B27-positive SpA patients and HCs (*P* = 0.69) or between HLA-B27-negative SpA patients and HLA-B27-positive SpA patients (*P* = 0.09).

To clarify whether shedding of CD18 relates to disease activity in SpA, we correlated shedding of CD18 from SpA patient-derived PBMCs with disease activity scores. The sCD18 concentrations in SpA PBMC supernatants correlated negatively with the BASDAI score (Figure 4B; *r* = -0.71, *P* <0.05). A similar trend of negative correlation with the sCD18 plasma concentration was found for the other disease scores recorded in this study (data not shown). Since CD18 is a substrate for MMP-9, we measured the concentration of MMP-9 in the same PBMC supernatants. In this case, the analysis presented a non-significant tendency of increasing sCD18 levels with increasing MMP-9 in supernatant from SpA PBMCs (Figure 4C; *r* = 0.58, *P* = 0.08). Taken together, these data suggest that insufficient generation of sCD18 in SpA could be part of an increased disease activity.

### CD18 was shed primarily from monocytes

**Figure 5:**
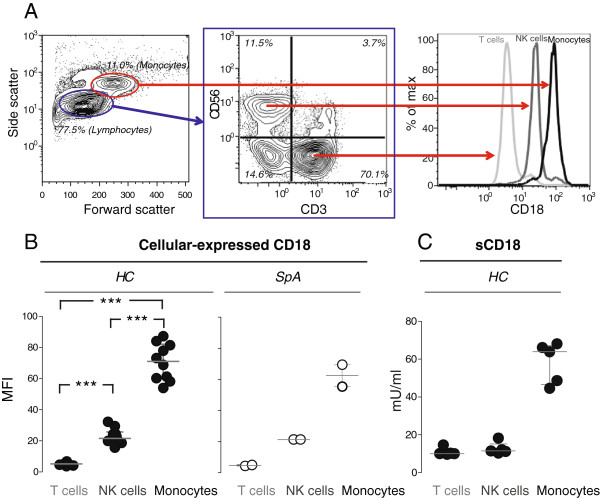
**Cellular-expressed and shed CD18 attributed to peripheral blood mononuclear cell (PBMC) source. (A)** The cell membrane expression of CD18 on T cells, natural killer (NK) cells, and monocytes using PBMCs from a representative healthy control (HC) with the gating strategy indicated. **(B)** The cell membrane expression of CD18 on T cells, NK cells, and monocytes using PBMCs from 10 HCs and 2 patients with spondyloarthritis (SpA). Levels of CD18 expression were measured by the median fluorescence intensity (MFI). Bars indicate median and interquartile range. **(C)** The concentration of sCD18 in supernatants from cultured T cells, NK cells, and monocytes derived from PBMCs from 5 HCs. Bars indicate median and interquartile range. ***P <0.001.

To more clearly identify the cellular sources of sCD18, the cell membrane expression and *in vitro* shedding were studied with HC- and SpA-derived PBMCs. For both HC- and SpA-derived PBMCs, the cell surface expression of CD18 was higher on monocytes compared with both T cells and NK cells as judged from the median fluorescence intensity (MFI). NK cells also showed a higher cell surface expression of CD18 compared with T cells (Figure 5A and B). The MFIs of the isotype control IgG1 staining were 0.406, 0.414, and 2.46 for T cells, NK cells, and monocytes, respectively. To study the capacity for shedding by these subsets, the cells were separated by application of antibodies to the canonical markers CD14, CD3, or CD56 of monocytes, T cells, and NK cells, respectively. The median percentages of CD14^+^ cells were 84.3% (interquartile range 82.8%-89.8%) in the isolated monocytes, 97.5% (interquartile range 96.6%-97.7%) of CD3^+^ cells in the isolated T cells, and 85.9% (interquartile range 84.1%-90.7%) of CD56^+^ cells in the isolated NK cells. As expected from the high CD18 cell surface expression, supernatants from monocyte cultures contained a higher sCD18 concentration than supernatants from both T cells and NK cells (Figure 5C).

**Figure 6:**
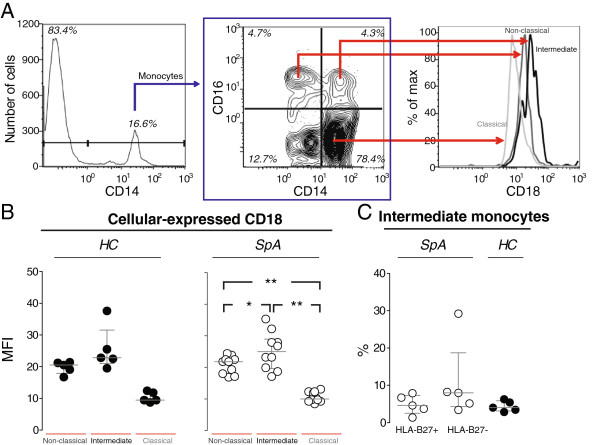
**CD18 expression and distribution of monocyte subsets in healthy controls (HCs) and patients with spondyloarthritis (SpA). (A)** The cell membrane expression of CD18 on CD14^+^ CD16^++^ (non-classic) monocytes, CD14^++^ CD16^+^ (intermediate) monocytes, and CD14^++^ CD16^-^ (classic) monocytes using peripheral blood mononuclear cells (PBMCs) from a representative HC with the gating strategy indicated. **(B)** The cell membrane expression of CD18 on non-classic monocytes, intermediate monocytes, and classic monocytes in PBMCs from 5 HCs and 10 patients with SpA. Bars indicate median and interquartile range. **(C)** The percentages of intermediate monocytes among all CD14^+^ monocytes in HLA-B27-positive SpA, HLA-B27-negative SpA, and HCs. PBMCs from the same 10 patients with SpA and 5 HCs were used. Bars indicate median and interquartile range. *P <0.05; **P <0.01; ****P <0.0001.

**Figure 7:**
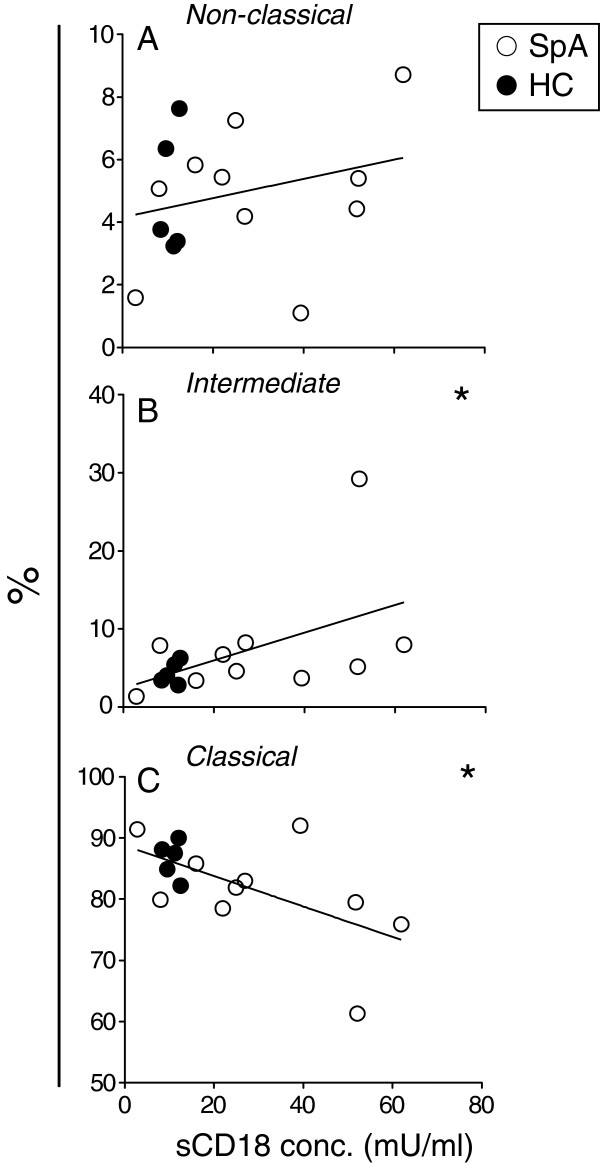
**Correlation between monocyte subsets and soluble CD18 (sCD18) in the peripheral blood mononuclear cell (PBMC) culture supernatant.** The percentage of non-classic **(A)**, intermediate **(B)**, or classic **(C)** monocytes among all monocytes in a sample of PBMCs was correlated against the sCD18 concentration in supernatant from culture of the PBMCs. Samples were analyzed from PBMCs derived from a total of 5 healthy controls (HCs) and 10 patients with spondyloarthritis (SpA).

Since monocytes had the highest CD18 cell membrane expression and showed the highest degree of CD18 shedding, we studied the monocyte subpopulations in further detail. Based on past investigations, the CD14^+^ cells were stratified into subsets of classic, intermediate, and non-classic monocytes by additional staining for cell membrane expression of CD16. The expression of CD18 was greater on CD14^++^ CD16^+^ (intermediate) monocytes compared with both CD14^+^ CD16^+^ (non-classic) monocytes and CD14^++^ CD16^-^ (classic) monocytes and was greater on non-classic monocytes compared with classic monocytes (Figure 6A and B). The CD14^+^ cells were also additionally stained for cell membrane expression of CD163. The expression of CD18 was stronger on CD163^+^ than CD163^-^ monocytes (*P* <0.001). Confirming the finding from previous studies of arthritis [7-9], there were a non-significant, greater proportion of intermediate monocytes among all monocytes in HLA-B27-negative SpA patients compared with HCs (Figure 6C). A positive correlation was observed between the proportion of non-classic (Figure 7A) and intermediate (Figure 7B) monocytes among all CD14^+^ monocytes and the concentration of sCD18 in the PBMC culture supernatants, whereas a negative correlation was found for the proportion of classic monocytes (Figure 7C).

## Discussion

The inflammatory process in SpA is still poorly elucidated, resulting in difficulties in monitoring disease activity and choosing treatment strategy. Several investigations in experimental animal models already indicate a central role of CD18 integrins in SpA-like disease [14-16,37]. We previously demonstrated alterations in the sCD18 complexes in the blood and synovial fluid of patients with arthritis and the ability of sCD18 to inhibit leukocyte binding to ICAM-1. This study proposes sCD18 as a novel player in SpA disease pathogenesis. The sCD18 plasma level was decreased in patients with SpA compared with HCs, and levels correlated inversely with SpA disease activity scores. The failure of patients with SpA to maintain normal sCD18 levels seems to reflect insufficient CD18 shedding from monocytes to counterbalance the capture of sCD18 complexes to inflammation-induced ICAM-1. In this way, our findings on sCD18 link increased leukocyte infiltration potential with disease activity in SpA.

As noted by Feldmann and colleagues [38], inflammation is regulated by the balance between pro- and anti-inflammatory mechanisms. In this sense, the inflammatory response may originate as a consequence of over-activity of pro-inflammatory mechanisms or mechanistic failures in anti-inflammatory responses. Previously, it was rather speculative to assign the production of sCD18 to either one of the two modes of regulating the inflammatory response. Yet the ability of sCD11/CD18 complexes to compete with cellular-expressed CD11a/CD18 in ICAM-1 binding [18] indicates that the soluble complexes may aid in resolving cellular adhesion and hence the formation of inflammatory foci. We now found at least two indications to support such anti-inflammatory functions of sCD11/CD18.

First, we demonstrated the ability of the sCD11/CD18 complexes to bind ICAM-1 in the cell membrane of a human umbilical vein cell line and primary FLSs established from a site of major inflammation in SpA. ICAM-1 expression is increased on the lining and sublining cells of the synovium and on adjacent endothelial cells in patients with SpA and is regulated by TNFα [39,40]. Our immunocytological staining for ICAM-1 showed a speckled pattern very similar to previous findings [35,36]. Carman and colleagues [35,36] revealed that the origin of this morphology is caused at least partly by the formation of ICAM-1-enriched cell membrane protrusions. These protrusions are critical in the pro-active role of the endothelium in the transmigration of leukocytes [35,36]. The deposition of sCD11/CD18 complexes on the cells investigated in our experiment was not uniform and appeared in a way that could well reflect the selective binding to such ICAM-1-enriched zones. Indeed, the deposition was critically dependent on TNFα treatment of the cells, increasing the ICAM-1 expression. The requirement of a dense expression of ICAM-1 is furthermore consistent with the biochemical evidence reported earlier [18] that multimers of sCD11/CD18 (for example, 2 × CD11/CD18 and 4 × CD11/CD18) participate in a polyvalent interaction with ICAM-1 molecules, enabling a high avidity in the interaction [18,41]. In this way, our findings support that sCD11/CD18 selectively binds ICAM-1-rich features important for the interaction of leukocytes with the synovial lining and endothelium of blood vessels. Likewise, the clinical investigations made in our study suggest that insufficient saturation of these features with sCD11/CD18 captured from the plasma pool may aggravate inflammation, probably by permitting undue leukocyte adhesion.

Second, it is an important observation that the negative correlation between the decreased plasma level of sCD18 and disease activity in SpA could be simulated in cell cultures *in vitro*. Thus, CD18 shedding from SpA patient-derived PBMCs correlated negatively with the BASDAI score. This indicates that inflammation may be downregulated by the ability of leukocytes to deliver an elevated production of sCD18. In contrast, failure by the leukocytes to compensate the capture of sCD11/CD18 onto ICAM-1-expressing surfaces appears to augment inflammation. Taken together, these data also indicate that a compound resembling sCD18 could be beneficial as a therapeutic drug in SpA.

Our investigations identified monocytes as the predominant contributors to CD18 shedding among PBMCs in cultures *in vitro*. It is well established that the monocyte population can be separated into subsets with distinct regulatory capabilities [5,42,43]. Intermediate monocytes have both pro-inflammatory and anti-inflammatory functions. Thus, they are precursors of osteoclasts in psoriatic arthritis [7] and promote the expansion of the Th17 subset in RA [8]. However, intermediate monocytes also show the highest expression of the anti-inflammatory cytokine IL-10 and are tolerant to stimulation during infection [6,44]. Also, lack of CD16^+^ monocytes may be related to auto-inflammatory disease [45]. In line with previous studies, we found a high expression of CD18 integrins in the membrane of monocytes and especially the intermediate monocyte subset [46,47]. Furthermore, the concentration of sCD18 in PBMC supernatants correlated with the percentage of intermediate monocytes among all monocytes. This suggests that intermediate monocytes are the primary producers of sCD18 in the PBMC subset. In this way, our study proposes a novel role for intermediate monocytes in the blood as regulators of leukocyte migration. In SpA this regulatory role seems to be inadequate. Thus, in HLA B27-negative SpA only, the percentage of intermediate monocytes among all monocytes was increased, resulting in sufficient upregulation of CD18 shedding and low disease activity. However, this increase in intermediate monocytes was not seen in HLA B27-positive SpA, resulting in insufficient CD18 shedding and higher disease activity.

The high degree of overlap between plasma levels of sCD18 in patients with SpA and HCs implies that measuring sCD18 will not be of particular value in the diagnosis of SpA. However, measuring sCD18 could assist in discriminating between inflammatory activity and broader problems due to chronic changes in SpA. From a clinical viewpoint, discrimination between ongoing inflammation and chronicity is thus critical for choosing the proper clinical intervention [2]. To best measure the level of inflammation, many composite scores, such as the BASDAI, BASFI, and physician global assessment score, have been developed. However, these measures are only moderately objective and are difficult to compare across individuals. Also, BASDAI does not correlate well with objective measures such as CRP and MRI findings or erythrocyte sedimentation rate [48]. Nevertheless, in lieu of better measures of disease activity, the BASDAI is currently regarded as the gold standard regarding treatment initiation and response [49]. This underlines the need for better objective measures, especially because the role of CRP in SpA is debatable, as it is induced mainly by IL-6 and anti-IL-6 has little effect in SpA [50,51]. In agreement, CRP showed no correlations with self-assessment scores, clinical findings, or MRI activity scores in our study population (data not shown). By contrast, the decrease in plasma levels of sCD18 found here correlated with a number of single and composite inflammatory measures, including BASDAI, level of morning stiffness, and physician global assessment score and the objective measures BASMI and SIJ MRI activity score in patients with SpA. These associations were greater when adjusting for differences in age, disease duration, HLA-B27 status, and treatment at time of inclusion, making the study population more homogeneous. Additionally, no correlation was observed between sCD18 and MRI chronicity scores or patient global score. Although MRI is not considered the best measure of structural damage, the MRI chronicity scores used in this study were based on MRI features described previously to correlate with radiographic changes [30,31]. Thus, this adds to the validity of sCD18 as a marker of the inflammatory component of SpA disability. Importantly, the association with disease activity remained after correction for CRP.

Recently, the serum concentrations of both MMP-3 and MMP-9 have been found to be associated with disease activity in ankylosing spondylitis [52,53]. Shedding of CD18 occurs in processes that involve MMP-3 and MMP-9 [22,23]. With the observation that CD18 is a substrate for MMP-9 [23], measurement on sCD18 may provide an indication of enzymatic activity *in vivo*, which is central to pathogenic mechanisms of these diseases [25]. In this way, sCD18 could be a novel mechanistic biomarker of SpA disease activity involved in the disease pathogenesis and reflecting inflammatory processes [54,55].

No inflammatory disease controls were assessed in this study. Thus, the specificity of our findings in SpA is uncertain. Interestingly though, the plasma levels of sCD18 were decreased in plasma from HLA-B27-positive SpA patients compared with HLA-B27-negative patients. Additionally, shedding of CD18 from PBMCs was increased among HLA-B27-negative patients only. HLA-B27 positivity in patients with ankylosing spondylitis predicts a poorer prognosis and a better response to anti-TNFα treatment [56]. It is unclear whether this reflects a different pathogenesis in HLA-B27-positive versus HLA-B27-negative SpA. HLA-B27-positive SpA patients also had increased BASMI, CRP, and MRI activity scores compared with HLA-B27-negative SpA patients in our study population (data not shown). From the established correlations, the decreased levels of sCD18 in HLA-B27-positive SpA may thus be explained by the higher degree of disease activity in this group. However, mechanistic couplings between HLA-B27 and shedding of CD18 cannot be excluded. Validation studies are needed to clarify whether measuring the sCD18 plasma level can be used as a marker of inflammatory activity in SpA.

## Conclusions

The clinical impact of CD18 deficiency has been long known from the several types of leukocyte adhesion deficiencies [57]. Our work now argues that more subtle changes in CD18 functions are sufficient to affect disease activity in SpA through alterations in the systemic concentration of sCD18. The influences appear to involve the failure of monocyte subsets to maintain a sufficient concentration of sCD11/CD18 complexes. Our work suggests that a delicate balance exists between cellular-expressed and sCD18 integrins, which may be disturbed by the well-characterized changes in MMP activity and ICAM-1 expression associated with a vast range of illnesses involving chronic inflammation. With the central role of CD18 integrins in supporting leukocyte migration and immunological synapse formation, it encourages the hypothesis that such disturbances are involved in the disease-causing mechanisms in SpA. This points to the level of sCD18 as a potential marker of inflammatory activity or a compound resembling sCD18 as a therapeutic drug.

## Abbreviations

BASDAI: Bath Ankylosing Spondylitis Disease Activity Index; BASFI: Bath Ankylosing Spondylitis Functional Index; BASMI: Bath Ankylosing Spondilitis Metrology Index; CR: complement receptor; CRP: C-reactive protein; DMEM: Dulbecco’s modified Eagle’s medium; FCS: fetal calf serum; FITC: fluorescein isothiocyanate; FLS: fibroblast-like synoviocyte; HC: healthy control; ICAM-1: intercellular adhesion molecule 1; mAb: monoclonal antibody; MFI: mean fluorescence intensity; MMP: matrix metalloproteinase; MRI: magnetic resonance imaging; NHS: normal human serum; NK: natural killer; PBMC: peripheral blood mononuclear cell; RA: rheumatoid arthritis; RT: room temperature; sCD18: soluble CD18; SFMC: synovial fluid mononuclear cell; SIJ: sacroiliac joint; SpA: spondyloarthritis; TBS: Tris-buffered saline; Th17: T helper 17; TNF-α: tumor necrosis factor-alpha; TRIFMA: time-resolved immunfluorometric assay; VAS: visual analogue scale.

## Competing interests

The authors declare that they have no competing interests.

## Authors’ contributions

TWK helped to design the study, to carry out the experiments, and to analyze and interpret the data and drafted the manuscript. TV-J helped to design the study and to analyze and interpret the data and was involved in revising the manuscript. BD helped to design the study, to collect the patient samples and information, and to analyze and interpret the data and was involved in revising the manuscript. BJ and AK helped to carry out the experiments and to analyze and interpret the data and were involved in revising the manuscript. BS-C and AGJ helped to collect the patient samples and information and to analyze and interpret the data and were involved in revising the manuscript. MH, RØ, and WHR helped to analyze and interpret the data and were involved in revising the manuscript. All authors read and approved the final manuscript.

## Supplementary Material

Additional file 1: Table S1 Associations at time of inclusion between plasma soluble CD18 (sCD18) levels in all patients with spondyloarthritis (SpA) and self-assessment scores after correction for age, disease duration, HLA-B27 status, treatment, and C-reactive protein (CRP). Click here for file

Additional file 2: Table S2 Associations at time of inclusion between plasma soluble CD18 (sCD18) levels in all patients with spondyloarthritis (SpA) and clinical scores and test results after correction for age, disease duration, HLA-B27 status, treatment, and C-reactive protein (CRP). Click here for file

Additional file 3: Figure S1 Confocal microscopy analysis of the ability of sCD11/CD18 complexes to bind intercellular adhesion molecule 1 (ICAM-1) expressed on the human umbilical vein cell line EA.hy926 or spondyloarthritis (SpA) fibroblast-like synoviocytes (FLSs). **(A)** Schematic of the cellular incubations. In step 1, adherent cells were incubated with 10 ng/mL tumor necrosis factor-alpha (TNFα), which increased the ICAM-1 expression. In step 2, a source of CD11/CD18 was added (that is, either NHS or supernatant from peripheral blood mononuclear cell (PBMC) culture). In step 3, biotinylated antibody recognizing ligand-binding activated CD11/CD18 (KIM127) was added followed by addition of fluorochrom-labelled streptavidin for detection with confocal microscopy. **(B)** Binding of soluble CD18 (sCD18) to TNFα-treated cells. As outlined above, in step 1, cells were treated with either TNFα or plain medium as a control. In step 2, either NHS or culture supernatant was used or plain medium was used as a control. In step 3, either the antibody to CD18 (KIM127) was used or biotinylated monoclonal IgG1 immunoglobulin was used as a control. Red staining indicated the binding of CD18, further indicated with white arrows. The positions of cell nuclei were located by 4′,6-diamidino-2-phenylindole (DAPI) staining, indicated in blue. Click here for file

Additional file 4: Figure S2 Depletion of soluble CD18 (sCD18) by binding to intercellular adhesion molecule 1 (ICAM-1) expressed on the human umbelical vein cell line EA.hy926 or spondyloarthritis (SpA) fibroblast-like synoviocytes (FLSs). Culture medium supplemented with 50% (vol/vol) normal human serum (NHS) or 50% (vol/vol) synovial fluid mononuclear cell (SFMC) supernatant as sCD18 source were incubated with EA.hy926 or SPA FLS cells, each cell type either cultured in the supplemented media with 10 ng/mL tumor necrosis factor-alpha (TNFα) (to induce ICAM-1 expression) or in the supplemented media without further additions (“Medium”). Click here for file
