# Supplementary material for: Decreased plasma levels of soluble CD18 link leukocyte infiltration with disease activity in spondyloarthritis
Source: Arthritis Res Ther. 2014 Feb 4;16(1):R42. doi: 10.1186/ar4471 (PMC3978678; doi:10.1186/ar4471)
Supplement: Additional file 1: Table S1 — Associations at time of inclusion between plasma soluble CD18 (sCD18) levels in all patients with spondyloarthritis (SpA) and self-assessment scores after correction for age, disease duration, HLA-B27 status, treatment, and C-reactive protein (CRP). [file ar4471-S1.doc]

**Table S1.**

Associations at time of inclusion between plasma sCD18 levels in all SpA patients and self-assessment scores after correcting for age, disease duration, HLA-B27 status, treatment and CRP.

|  |  | **BASDAI** | **BASFI** | **Patient pain** | **Patient global** | **Morning stiffness (level)** | **Morning stiffness (duration)** |
| --- | --- | --- | --- | --- | --- | --- | --- |
| **sCD18** | r^a^  *P* | **-0.32**  **0.014** | -0.093  0.49 | -0.14  0.29 | -0.12  0.40 | **-0.30**  **0.024** | -0.18  0.18 |

Bold numbers indicate significant correlations. ^a^ Partial correlation coefficients.
