# Supplementary material for: Decreased plasma levels of soluble CD18 link leukocyte infiltration with disease activity in spondyloarthritis
Source: Arthritis Res Ther. 2014 Feb 4;16(1):R42. doi: 10.1186/ar4471 (PMC3978678; doi:10.1186/ar4471)
Supplement: Additional file 2: Table S2 — Associations at time of inclusion between plasma soluble CD18 (sCD18) levels in all patients with spondyloarthritis (SpA) and clinical scores and test results after correction for age, disease duration, HLA-B27 status, treatment, and C-reactive protein (CRP). [file ar4471-S2.doc]

**Table S2.**

Associations at time of inclusion between plasma sCD18 levels in all SpA patients and clinical scores and test results after correcting for age, disease duration, HLA-B27 status, treatment and CRP.

|  |  | **BASMI** | **Physician global** | **Thoracic chest expansion** | **SIJ activity** | **Spine activity** |
| --- | --- | --- | --- | --- | --- | --- |
| **sCD18** | r^a^  *P* | **-0.29**  **0.022** | **-0.36**  **0.0048** | 0.14  0.26 | -0.23  0.066 | -0.029  0.85 |

Bold numbers indicate significant associations. ^a^ Partial correlation coefficients.
