# Supplementary figures and images for: Decreased plasma levels of soluble CD18 link leukocyte infiltration with disease activity in spondyloarthritis
Source: Arthritis Res Ther. 2014 Feb 4;16(1):R42. doi: 10.1186/ar4471 (PMC3978678; doi:10.1186/ar4471)

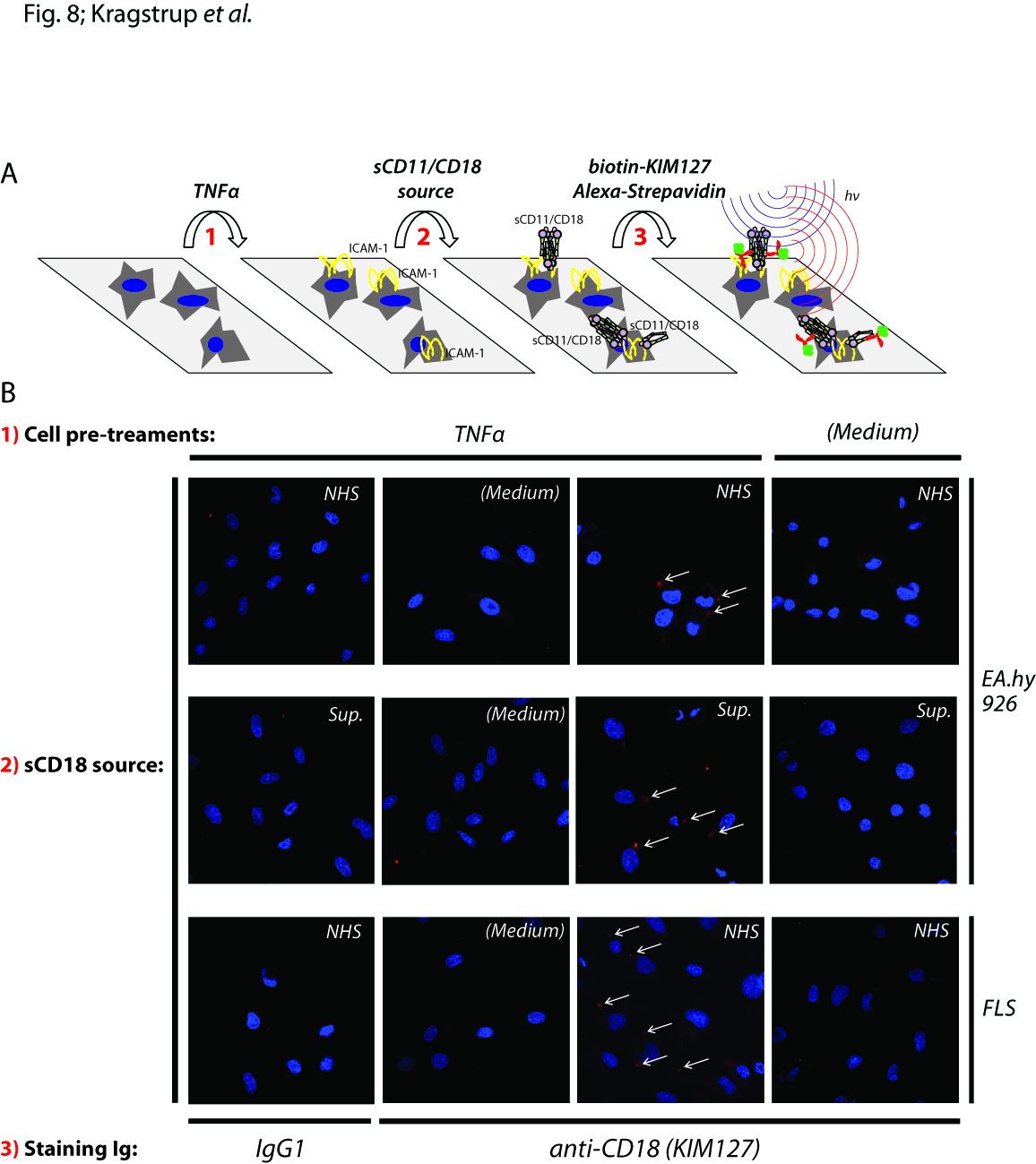

Supplement: Additional file 3: Figure S1 — Confocal microscopy analysis of the ability of sCD11/CD18 complexes to bind intercellular adhesion molecule 1 (ICAM-1) expressed on the human umbilical vein cell line EA.hy926 or spondyloarthritis (SpA) fibroblast-like synoviocytes (FLSs). (A) Schematic of the cellular incubations. In step 1, adherent cells were incubated with 10 ng/mL tumor necrosis factor-alpha (TNFα), which increased the ICAM-1 expression. In step 2, a source of CD11/CD18 was added (that is, either NHS or supernatant from peripheral blood mononuclear cell (PBMC) culture). In step 3, biotinylated antibody recognizing ligand-binding activated CD11/CD18 (KIM127) was added followed by addition of fluorochrom-labelled streptavidin for detection with confocal microscopy. (B) Binding of soluble CD18 (sCD18) to TNFα-treated cells. As outlined above, in step 1, cells were treated with either TNFα or plain medium as a control. In step 2, either NHS or culture supernatant was used or plain medium was used as a control. In step 3, either the antibody to CD18 (KIM127) was used or biotinylated monoclonal IgG1 immunoglobulin was used as a control. Red staining indicated the binding of CD18, further indicated with white arrows. The positions of cell nuclei were located by 4′,6-diamidino-2-phenylindole (DAPI) staining, indicated in blue. [file ar4471-S3.tiff]

Fig. S2; Kragstrup *et al.*

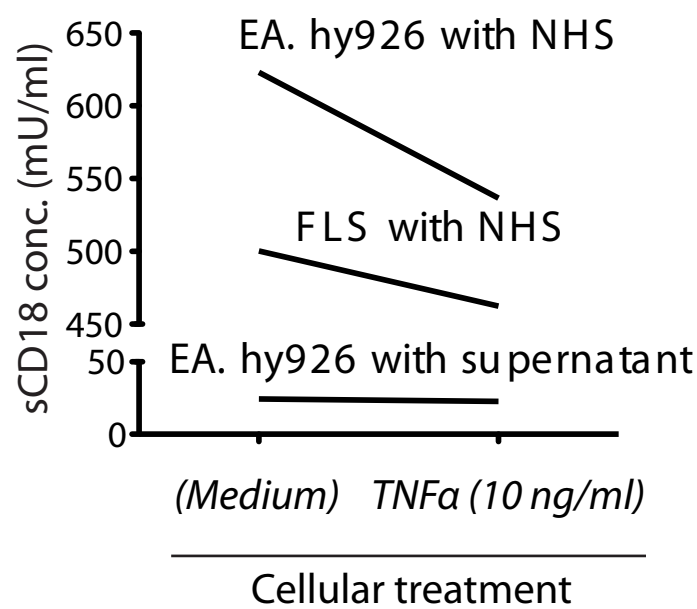

Supplement: Additional file 4: Figure S2 — Depletion of soluble CD18 (sCD18) by binding to intercellular adhesion molecule 1 (ICAM-1) expressed on the human umbelical vein cell line EA.hy926 or spondyloarthritis (SpA) fibroblast-like synoviocytes (FLSs). Culture medium supplemented with 50% (vol/vol) normal human serum (NHS) or 50% (vol/vol) synovial fluid mononuclear cell (SFMC) supernatant as sCD18 source were incubated with EA.hy926 or SPA FLS cells, each cell type either cultured in the supplemented media with 10 ng/mL tumor necrosis factor-alpha (TNFα) (to induce ICAM-1 expression) or in the supplemented media without further additions (“Medium”). [file ar4471-S4.pdf]
